# Cytokine Modification of Adoptive Chimeric Antigen Receptor Immunotherapy for Glioblastoma

**DOI:** 10.3390/cancers15245852

**Published:** 2023-12-15

**Authors:** Kristen D. Pawlowski, Joseph T. Duffy, Stephen Gottschalk, Irina V. Balyasnikova

**Affiliations:** 1 Department of Neurological Surgery, Wake Forest School of Medicine, Winston-Salem, NC 27101, USA; kraue@wakehealth.edu; 2 Department of Neurological Surgery, Northwestern University, Chicago, IL 60208, USA; joseph.duffy1@northwestern.edu; 3 Northwestern Medicine Malnati Brain Tumor Institute, Lurie Comprehensive Cancer Center, Feinberg School of Medicine, Northwestern University, Chicago, IL 60208, USA; 4 Department of Bone Marrow Transplantation and Cellular Therapy, St. Jude Children’s Research Hospital, Memphis, TN 38105, USA; stephen.gottschalk@stjude.org

**Keywords:** CAR T-cell, CAR NK-cell, cytokine, chemokine, glioblastoma

## Abstract

**Simple Summary:**

Chimeric antigen receptor (CAR) cell-based therapy is a promising treatment approach for glioblastoma, a fatal brain cancer with limited treatment options. Efforts to improve these therapies are currently underway, with one method being cytokine modification. Cytokines are a type of protein created by cells in the body that can change the function of immune cells. In this review, the authors summarize approaches that employ cytokines to improve CAR cell therapies. These include coadministering CAR T-cells or CAR NK-cells with cytokines, antibodies, or oncolytic viruses or via engineering CAR cell therapies to secrete or express cytokines, express a cytokine receptor, or genetically alter cytokine signaling pathways. We hope that providing cytokine support to CAR cell-based therapies will improve their efficacy for patients with glioblastoma.

**Abstract:**

Chimeric antigen receptor (CAR) cell-based therapies have demonstrated limited success in solid tumors, including glioblastoma (GBM). GBMs exhibit high heterogeneity and create an immunosuppressive tumor microenvironment (TME). In addition, other challenges exist for CAR therapy, including trafficking and infiltration into the tumor site, proliferation, persistence of CARs once in the tumor, and reduced functionality, such as suboptimal cytokine production. Cytokine modification is of interest, as one can enhance therapy efficacy and minimize off-target toxicity by directly combining CAR therapy with cytokines, antibodies, or oncolytic viruses that alter cytokine response pathways. Alternatively, one can genetically modify CAR T-cells or CAR NK-cells to secrete cytokines or express cytokines or cytokine receptors. Finally, CARs can be genetically altered to augment or suppress intracellular cytokine signaling pathways for a more direct approach. Codelivery of cytokines with CARs is the most straightforward method, but it has associated toxicity. Alternatively, combining CAR therapy with antibodies (e.g., anti-IL-6, anti-PD1, and anti-VEGF) or oncolytic viruses has enhanced CAR cell infiltration into GBM tumors and provided proinflammatory signals to the TME. CAR T- or NK-cells secreting cytokines (e.g., IL-12, IL-15, and IL-18) have shown improved efficacy within multiple GBM subtypes. Likewise, expressing cytokine-modulating receptors in CAR cells that promote or inhibit cytokine signaling has enhanced their activity. Finally, gene editing approaches are actively being pursued to directly influence immune signaling pathways in CAR cells. In this review, we summarize these cytokine modification methods and highlight any existing gaps in the hope of catalyzing an improved generation of CAR-based therapies for glioblastoma.

## 1. Introduction

Glioblastoma (GBM) is a fatal primary brain tumor with limited therapeutic options. The standard of care treatment for maximal surgical resection, radiation, and temozolomide chemotherapy extends survival by months [1,2]. The success of chimeric antigen receptor (CAR) T-cell or natural killer (NK)-cell therapies in hematological malignancies [3,4,5,6,7] has led to extensive efforts for translation in solid tumors, including GBM [8,9]. CAR T-cell therapies in GBM have been designed to target numerous tumor-specific antigens, such as epidermal growth factor receptor variant III (EGRFvIII) [10,11,12,13], interleukin13 receptor alpha 2 (IL13Rα2) [14,15,16,17,18], human epidermal growth factor receptor 2 (HER2) [19,20], B7-H3 (CD276) [21,22,23], disialoganglioside (GD-2) [24], and erythropoietin-producing hepatocellular carcinoma A 2 (EphA2) [25]. Despite promising preclinical results, CAR cell-based therapies have limited efficacy in early-phase clinical studies [26,27]. This failure is primarily attributed to high target antigen heterogeneity, the emergence of antigen-loss variants, the presence of glioma stem cells, poor tumor infiltration, persistence, and proliferation of CAR T-cells or CAR NK-cells, and suboptimal cytokine secretion upon CAR cell stimulation [28,29]. Additionally, CAR T-cell therapies in GBM have been associated with life-threatening toxicities such as cytokine release syndrome (CRS), immune effector cell-associated neurotoxicity syndrome (ICANS), and tumor inflammation-associated neurotoxicity (TIAN), all of which have been associated with the activation of endogenous immune cells and the cytokines they secrete [27,29,30,31,32,33].

**Figure 1:**
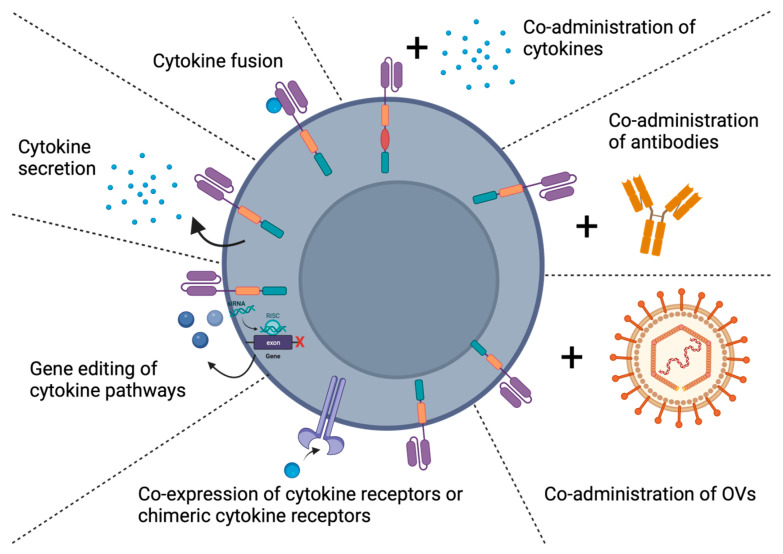
Cytokine modification techniques to enhance CAR cell-based therapies for glioblastoma. Chimeric antigen receptor (CAR) cell-based therapies have had limited success in solid tumors such as glioblastoma (GBM). Multiple strategies have been utilized to enhance CAR T or natural killer (NK) cells against GBM tumors, which include coadministration of cytokines, antibodies, or oncolytic viruses (OVs) or through genetically engineering CARs to secrete cytokines, express cytokines fused to the CAR, display cytokine receptors/chimeric cytokine receptors, or to augment/suppress cytokine pathways directly. Created with BioRender.com.

Methods to improve CAR cell-based therapy for GBM are currently underway and include multiantigen targeting, gene knock-out and knock-in techniques, controlled or inducible CAR expression systems, and cytokine modifications [28]. Modifying cytokines is particularly interesting, as it may improve the efficacy and reduce the toxicity of CAR T-cell or CAR NK-cell therapies. Methods for cytokine modification include combination therapy of CARs with locally delivered cytokines, antibodies, or oncolytic viruses designed to stimulate or inhibit cytokine function. Alternatively, CAR T-cells or CAR NK-cells can be built to secrete or express cytokines, cytokine receptors, or chimeric cytokine receptors, or be genetically modified to suppress cytokine pathways (Figure 1). While several excellent reviews on the topic of CAR cell therapy for GBM have recently been published [34,35,36,37,38,39,40,41,42], to our knowledge, none has specifically focused on cytokine modifications of immune cells. Likewise, our review is not only relevant to CAR cell therapies for GBM, but also for other cancers for which CAR cell therapies are currently being developed.

## 2. Coadministration of Cytokines

T-cell activation and expansion require three distinct signals: antigen-specific activation, costimulation, and cytokine-mediated differentiation and expansion. While CARs can be readily designed to provide antigen-specific activation and costimulation, CAR-induced cytokine secretion by T-cells is limited in the immunosuppressive environment of the TME [43]. Coadministering immunostimulatory cytokines alongside CAR T-cells is the most straightforward way to enhance treatment efficacy and has been successfully used in many solid tumors, including melanoma and ovarian cancer [44,45,46]. This can be performed either systemically through intravenous injection or intracranially through surgical techniques. Cytokines such as IL-2 have been used to treat melanoma patients for decades, and the benefit of combination treatment with other immunotherapies has been well-established in randomized clinical trials for years [47,48,49,50,51,52]. 

Progress has been relatively slow compared to glioblastoma, possibly due to its greater immunosuppressive profile and lack of robust clinical benefit with cytokine monotherapy [53]. Recent efforts to combine cytokine therapy with CAR immunotherapy are currently underway. For example, Agilardi and colleagues recently developed a method for delivering IL-12 alongside EGFR-directed CAR T-cells to enhance their activity [54]. EGFRvIII-CAR T-cells, while promising in preclinical studies, have not shown benefit when tested in humans [13]. Agilardi and colleagues recognized that IL-12 is a potent proinflammatory cytokine that improves T-cell effector function [55]; however, it has severe toxicity when administered systemically [55,56]. They, therefore, modified the cytokine so that it is attached to the Fc portion of murine IgG3 to enhance the cytokine [54]. This modified IL-12 was then introduced intratumorally via stereotactic injection into the tumor bed following the intravenous delivery of EGFRvIII-CAR T-cells. As anticipated, this combination significantly reduced tumor burden, decreased exhaustion markers on CAR T-cells, and had a synergistic outcome on overall survival in a mouse model of GBM. Upon further investigation, the authors discovered that the enhancement of CAR T-cell therapy was not due to increased infiltration but rather to prevent CAR T-cell dysfunction and promote CAR T-cell-derived cytokines like IFN-γ and TNF-α. Furthermore, local delivery of IL-12 did not cause transient elevations of serum IL-12, IL-6, and GM-CSF, all cytokines associated with CRS and ICANS [54]. It is reasonable, however, to remain skeptical that intracranial or systemic delivery of cytokines alongside CAR therapy would prove the best method to enhance CAR T or NK-cell function. Both methods rely on the pharmacokinetics of each cytokine, which may limit its therapeutic benefit. For example, the half-life of IL-2 is only 5–7 minutes [57]. This method, therefore, requires numerous administrations of the cytokine, making it more impractical for patients. If one is to investigate cytokine administration alongside CAR therapy, systemic therapy would be more prudent, given the toxicity associated with systemic administration.

## 3. Antibodies to Modulate Cytokine Function

Antibodies have been used to target cytokines involved in toxicities associated with CAR T-cell therapies. Briefly, antibodies can be designed to bind to a cytokine, which can block its ability to bind to a cytokine receptor, thus decreasing cytokine function. O’Rourke and colleagues, within a clinical trial testing an EGFR-based CAR T-cell therapy for glioblastoma, quantified cytokines in the peripheral blood of infused patients [13] (Table 1). Five of the ten subjects had a 10-fold or higher elevation in IL-6 concerning CRS. Methods to target IL-6 include the antibody tocilizumab, which targets the cytokine IL-6 receptor, and siltuximab, a monoclonal antibody against IL-6. Both antibodies have been used to treat patients experiencing moderate to severe CRS with or without corticosteroids [13,31,58,59,60]. It is important to note, however, that both Tocilizumab and Siltuximab can alter the measure of circulating IL-6 in patients, and thus, the assessment of recovery from CRS must then rely on clinical judgment of patient’s symptoms rather than laboratory values alone [60]. Nonetheless, anti-IL-6 treatment and corticosteroids remain the most used therapeutic strategies to modify cytokine levels for applying CAR T-cells in GBM.

While IL-6 is thought to largely play a role in CRS, the IL-6 blockade has little benefit in patients who develop ICANS or TIANS toxicities [33,59]. ICANS is managed with supportive care, corticosteroids, or the IL-1 receptor antagonist, anakinra [59,61]. TIANS, a more recently described toxicity in which inflammation occurs locally at the tumor site, resulting in the worsening of neurological function corresponding to nearby structures rather than global encephalopathy, has been managed similarly, either with supportive care, drainage of CSF, steroids, or with anakinra [33]. 

In addition to using monoclonal antibodies to combat CAR cell therapy toxicity, antibodies, such as those against PD-L1, have been used to augment CAR T-cell therapy’s antitumor effect. Excessively elevated levels of IFN-γ have been shown to induce PD-L1 (programmed death ligand 1) expression in tumor cells [62]. An et al. demonstrated that CAR T-cells designed against the Ephrin type-A receptor 2 (EphA2) receptor epitope B (EphA2-b) had an extremely high IFN-γ level compared to the epitope A (EphA2-a) variant and demonstrated higher PD-L1 surface expression levels on GBM cells and an overall lower effectiveness of tumor cell killing [63] (Table 1). The authors restored antitumor activity to similar levels seen in EphA2-a-CAR T-cells by using PD1 blockade therapy combined with EphA2-b-CAR T-cells. PD1 blockade did not affect EphA2-a-CAR T-cells. These results signify that modification of IFN-γ signaling via PD1 blockade can improve CAR T-cell therapy against EphA2-b-expressing gliomas, reiterating the importance of maintaining a delicate balance of cytokine levels for an effective therapeutic response.

Dong et al. demonstrated that anti-VEGF therapy can improve the function of EGFR-vIII-directed CAR T-cells [64] (Table 1). Previous reports indicate that EGFRvIII CAR T-cell therapy can only migrate to the invasive edges of GBM or limited areas of the TME [13]. Blocking VEGF has previously been demonstrated to normalize tumor vessels within the GBM [65]. The authors, therefore, sought to test if the addition of an antimouse monoclonal VEGF antibody (B20, Genentech) to EGFRvIII CAR T-cell therapy in immunocompetent mouse models bearing orthotopic GBM cell lines that express EGFRvIII, CT2A and GSC005, could improve tumor infiltration of CAR-T cells [64]. Combining anti-VEGF and EGFRvIII CAR T-cells enhanced survival and increased CAR T-cell accumulation intracranially by 11.5% compared to monotherapy in CT2A and GSC005 brain tumor xenograft models. Combination treatment also led to a more immunostimulatory TME, increasing CD8+ T-cell expression of granzyme B and TNFα and decreasing immunosuppressive FoxP3+ CD4 T-cell accumulation. Taken collectively, anti-VEGF therapies can promote CAR T-cell persistence and efficacy within an EGFR model of GBM.

Finally, it is important to mention that current clinical trials are investigating CAR therapy and antibodies designed to enhance cytokine function. For example, clinical trial NCT04003649 is actively recruiting participants to receive IL13Rα2-CAR T-cells alone or together with nivolumab (an antibody against the PD-L1 receptor) and ipilimumab (an antibody against CTLA-4), with an estimated completion December of 2024.

**Table 1:** Antibody approaches to modulate cytokine function in CAR therapy.

Tumor Antigen	CAR Effector Cell	Study Type	Animal Model	Antibody Cytokine Target	Delivery	TMZ, XRT, or Steroids	Results	Reference
EGFR	T-cell	C	N/A	Anti-IL6	IV	TMZ (p), XRT (p), steroids (p/c)	Improvement in CRS symptoms; inaccurate measure of circulating IL-6.	[13]
	T-cell	L	Mouse	Anti-VEGF (B20)	IV	No	Improved CAR T infiltration; prolonged survival; immunostimulatory effect in the TME.	[64]
EphA2-b	T-cell	L	Mouse	Anti-PD-L1	IC	No	Downregulation of IFN-γ to appropriate levels; increase in antitumor activity.	[63]
IL13Rα2	T-cell	C	N/A	Anti-PD-L1 and anti-CTLA-4	IT (CAR) and IV (antibodies)	TMZ (p), XRT (p), steroids (p/c)	N/A	NCT- 04003649

Abbreviations: C = clinical trial, L = preclinical laboratory, IV = intravenous, IC = intracranial, IT= intraventricular, EGFR = epidermal growth factor receptor, p = prior to CAR delivery, c = concurrent with CAR therapy, IL = interleukin, VEGF= vascular endothelial growth factor, TME= tumor microenvironment, EphA2 = ephrin type A receptor 2, PD-L1 = programmed cell death ligand 1.

## 4. Altering Cytokine Expression via Oncolytic Viruses

Oncolytic viruses (OV) represent an alternative form to modulate cytokine expression for CAR T-cells or CAR-NK-cells. OVs are viruses designed to selectively replicate in tumor cells, resulting in their lysis [66]. OVs can upregulate cytokine secretion by tumor cells and, thus, increase the therapeutic potential of CARs. Wang et al. designed OVs to include the chemotactic cytokine, or “chemokine”, CXCL11, and found that coadministration of OVs increases the therapeutic efficacy of B7-H3-CAR T-cells against GBM tumors [67] (Table 2). CXCL11 is a potent chemokine that recruits T-cells into the tumor microenvironment [68,69,70,71,72]. OV-CXCL11 increased migration and infiltration of B7-H3-CAR T-cells, as demonstrated using a Transwell migration assay containing U87-MG GBM cells [67]. The combination of B7-H3-CAR T-cells and OV-CXCL11 also showed superior cytotoxicity and greater production of proinflammatory cytokines (TNF-α, IFN-γ) in coculture with U87 cells. When assessed in vivo, the OV-CXCL11 and CAR T-cell combination further diminished tumor volume, significantly prolonged survival, increased CAR T-cell infiltration, and increased proinflammatory cytokine production (TNF-α and IFN-γ) compared to monotherapy alone. Furthermore, administration of OV-CXCL11 induced a greater abundance of NK-cells and M1-like macrophages and reduced the abundance of M2-like macrophages, T-regulatory cells, and MDSCs. In combining OV-CXCL11 therapy with B7-H3-CAR-T, the authors were able to simultaneously combat two main challenges of CAR T therapy against GBM—the immunosuppressive TME and insufficient infiltration of T-cells into the tumor site (Table 2). 

Zhu et al. generated an OV that induces IFN-γ secretion by CD70+ glioma cells to use with their CD70-CAR T-cells [73] (Table 2). In vitro, combination therapy killed most tumor cells at 24 hours and all tumor cells at 48 hours. When assessed in a PBMC-humanized NSG-B2m mouse model, combination therapy resulted in improved survival when compared to CD70-CAR T-cells on their own, with a single treatment of CD70-CAR T-cells and IFN-γ-OVs demonstrating complete regression in mice at day 35. Finally, the combination of IFN-γ-OVs and CAR T-cells altered the TME to be less immunosuppressive, showing an increase in NK-cell tumor infiltration, a decrease in T regulatory cells, and a significant decrease in TGF-β1 production.

Ma et al. generated an IL-15/IL15 receptor alpha (IL-15Rα) fusion OV (OV-IL15C) for combinatorial therapy with EGFR-CAR NK-cells for the treatment of GBM [66] (Table 2). In vitro treatment of two glioma cell lines, LN229 and U251, with EGFR-CAR NK-cells infected with OV-IL15C significantly increased IFN-γ and TNF-α production. In vivo, OV-IL15C and EGFR-CAR NK-cell therapy demonstrated a synergistic effect, reducing tumor burden post-treatment on day 15, prolonging survival, and generating 25% of long-term surviving mice (>50 days) in a GBM30 PDX mouse model. In an immunocompetent host, the combination of OV-IL15C and EGFR-CAR NK significantly enhanced endogenous NK and CD8+ T-cell infiltration, improved the persistence of EGFR-CAR NK-cells within the tumor site, and prolonged the survival of glioma-bearing mice. Finally, analysis of 37 cytokines, including IL-6, assessed with CAR NK monotherapy or combination therapy of OV-1L15C and EGFR-CAR NK-cells did not demonstrate significant differences in cytokine expression, suggesting a low risk of CRS.

IL-7 is known to augment the persistence of tumor-specific T-cells [74] and enhance the antitumor activity of T-cells [75,76]. To improve the efficacy of B7-H3-CAR T-cell therapy in glioblastoma, Huang et al. combined an IL-7-encoding OV (IL-7 OV) with B7-H3-CAR T-cells [77] (Table 2). When assessed as monotherapy, B7-H3-CAR T-cell therapy produces only moderate antitumor activity [21,22,23]. When B7-H3-CAR T-cells are combined with IL-7 OV, significantly improved CAR T-cell proliferation and enhanced killing of glioma cells are observed in vitro. When assessed in tumor-bearing mice, combination therapy showed considerably more significant numbers of tumor-infiltrating CAR T-cells and, thus, improved tumor regression and survival, with 4 out of 5 mice demonstrating long-term survival at day 60.

**Table 2:** Oncolytic viruses are designed to modulate cytokine or chemokine function in CAR therapy.

Tumor Antigen	CAR Effector Cell	Study Type	Animal Model	OV Construct	Delivery	TMZ, XRT, or Steroids	Results	Reference
B7-H3	T-cell	L	Mouse	CXCL11	IV (CAR) and IC (OV)	XRT	Increase in secretion of proinflammatory cytokines (TNF-α, IFN-γ); improved migration of CAR T-cells into the tumor; improvement of the TME immunosuppressive environment; prolonged survival and reduced tumor burden in both immunocompromised and immunocompetent mouse models	[67]
	T-cell	L	Mouse	IL-7	IV (CAR) and IC (OV)	No	Synergistic antitumor effect compared to monotherapy, augmented CAR T-cell proliferation.	[77]
EGFR	NK-cell	L	Mouse	IL15/IL15Rα	IC	No	Synergistic improvement of tumor burden and survival; enhanced endogenous immune function; improved persistence of therapy; no effect on exhaustion markers PDL-1 or Tim-3; low risk of CRS.	[66]
CD70	T-cell	L	Mouse	IFN-γ	IC (OV) and IV (CAR)	No	Improved tumor regression and survival; generated less immunosuppressive TME.	[73]

Abbreviations: C = clinical trial, L = pre-clinical laboratory, IV = intravenous, IC = intracranial, IT = intraventricular, CXCL11 = chemokine ligand 11, IL = interleukin, CAR = chimeric antigen receptor, TME = tumor microenvironment, NK = natural killer, PD-1 = program cell death receptor 1, CRS = cytokine release syndrome, CCL5 = chemokine C-C motif ligand 5, FRvIII = epidermal growth factor receptor variant III, GBM = glioblastoma, CD70 = clustered domain 70, IFN-γ = interferon gamma.

## 5. CARs Secreting or Expressing Cytokines

CARs designed to secrete cytokines have now emerged as an alternative strategy to increase the efficacy of CAR T-cells or CAR NK-cells against tumors. Zimmermann et al. genetically modified CAR T-cells targeting GD2+ gliomas with a nuclear factor of activated T cells (NFAT) inducible cytokine system [78] (Table 3). The viral vector encoded a constitutively expressed GD2-CAR and a synthetic NFAT enhancer/promoter (NFATsyn) to link transgenic IL-12 or IL-18 expression to T-cell activation. GD2-CAR/NFATsyn-IL-12 T-cells readily killed GD2+ patient-derived glioma spheroids and secreted IL-12, TNF-α, IFN-γ, Il-2, perforin, and granzyme B upon coculture with GD2+ glioma cells. Similar results were seen with IL-18-inducible CAR T-cells. Finally, both GD2-CAR/NFATsyn T-cell populations had enhanced cytolytic activity in vitro. The significant advantage of these inducible cytokine-producing CAR T-cells is their local therapeutic effects, maximizing on-tumor capability and diminishing the off-tumor, systemic toxicity. Furthermore, the group recently improved their manufacturing process to generate GD2-CAR/NFATsyn-IL-18 T-cells from two patients at a clinical scale and were not impeded by cryopreservation, demonstrating the feasibility of this treatment to reach patient care [79]. 

Rudek and Zimmermann et al. have also applied this technology to CAR-NK-cells [80] (Table 3). The authors utilized a nuclear factor kappa-light-chain enhancer of activated B cells (NFκB)—an inducible promoter—to link IL-12 cytokine production to NK-cell activation. When cocultured with GD2-positive gliomas, the modified GD2-CAR NK-cell exhibited less nonspecific killing of GD2-negative tumor cells. IL12-CAR NK-cells also demonstrated on-target specificity of IL-12 production when cocultured with GD2-positive tumor cells and did not produce IL-12 in the presence of GD2-negative tumor cells. They found that increased tumor killing also corresponded to an increase in cytolytic cytokines, such as IFN-γ, IL-2, IL-10, TNF-α, sFasL, and granzyme B.

Other studies have genetically modified CAR T cells to express multiple cytokines. Meister et al. generated natural killer group 2D (NKG2D)-targeted CAR T-cells that coexpressed the proinflammatory cytokines IL-12 and IFNα2 [81] (Table 3). Specifically, the CARs were generated via electroporation of the mRNAs encoding NKG2D or the cytokines. The authors evaluated these CAR T cells in three orthotopic immunocompetent mouse glioma models and found elevated proinflammatory TME, reduced T-cell exhaustion, and enhanced antitumor activity. This study illustrated the feasibility and efficacy of modifying CAR T-cells to express multiple cytokines.

Finally, our group has also generated IL13Rα2-directed CAR T-cells expressing IL-15 [82], a proinflammatory cytokine that stimulates activation and survival of T, NK, and NKT cells [83] (Table 3). These IL-15-enhanced CAR T-cells were generated using an IL-15-encoding retroviral vector by replacing the ΔCD34 gene in pSFG.iC9-2A-ΔCD34-2A-IL15 with a cytoplasmic domain-truncated nerve growth factor receptor gene (ΔNGFR) to generate the pSFG.iC9-2A-ΔNGFR-2A-IL15 construct. The transgenic expression of IL-15 induced more significant antigen-specific proliferation of CAR T-cells, enhanced production of cytokines, and improved survival in a glioma mouse model compared to the unmodified CAR T-cell construct. However, IL-15-modified CAR T-cells were still susceptible to antigen escape as gliomas that recurred after 40 days following CAR T-cell treatment, suggesting that targeting multiple antigens may still be necessary in addition to providing cytokine support. Similar results have been seen when designing CAR T-cells to secrete IL-15 within CAR T-cells targeting the glioblastoma antigens fibroblast growth factor-inducible 14 (Fn14) [84] and GD2 [85] (Table 3).

We also recently expanded upon these results to assess whether there is a difference in therapeutic efficacy between a CAR T-cell secreting the cytokine and expressing the cytokine as a fusion protein on the CAR T-cell [86] (Table 3). We found that while both strategies were efficacious in increasing CAR cell toxicity against glioblastoma tumors, the CAR T-cells that contained the fusion protein had superior antitumor results when assessed in vivo as well as improved survival within two immunocompetent GBM mouse models. These effects held even upon the rechallenge of therapy within long-term survivals, with the CAR T-cells modified with the IL-15 fusion protein having significantly prolonged survival compared to their IL-15-secreting counterparts. The effect may be because IL-15-fusion CAR T-cells resulted in a more significant effect on the TME via increasing immunostimulatory host CD8+ T-cells, NK-cells, and B-cells and decreasing suppressive host myeloid cells more effectively than IL-15-secreting CAR T-cells. Both strategies of designing CAR T-cells to either secrete or express a cytokine, however, were overall more beneficial than a CAR T-cell intended only to target a tumor antigen, supporting the argument that cytokine modifications can lead to an improved generation of CAR therapy within glioblastoma.

**Table 3:** Cytokine-secreting CAR constructs against GBM.

Tumor Antigen	CAR Effector Cell	Study Type	Animal Model	Cytokine	Delivery	TMZ, XRT, or Steroids?	Results	Reference
IL13Rα2	T-cell	L	Mouse	IL-15	IC	No	Improved antitumor activity, although subjected to antigen escape	[82]
	T-cell	L	Mouse	IL15s vs IL-15f	IC	No	IL-15 fusion to the CAR surface had superior function compared to CAR-secreting IL-15 in vitro and improved survival in two immunocompetent mouse models.	[86]
Fn14	T-cell	L	Mouse	IL-15	IV	No	Augmentation of antitumor effects of CAR T-cells, more prolonged remission, and survival in a mouse model.	[84]
GD2	T-cell	L	N/A	IL-12/IL-18	N/A	N/A	Increase in proinflammatory cytokines, enhanced cytotoxicity of CAR T-cells	[78]
	T-cell	L	Mouse	IL-15	IV	No	Improved survival and reduced tumor burden; no signs of neurotoxicity.	[85]
	NK-cell	L	N/A	IL-12	N/A	N/A	Less cytotoxicity compared to nonmodified CAR NK-cells was equivalent, but with more on-target specificity; target-specific IL-12 production was reserved for modified CAR NK-cells.	[80]
NKG2D	T-cell	L	Mouse	IL12, IFNα2	IV	N/A	Promoted a proinflammatory TME, reduced T-cell exhaustion, and enhanced anti-tumor activity.	[81]

Abbreviations: C = clinical trial, L = preclinical laboratory, IV = intravenous, IC = intracranial, IT = intraventricular, IL13Rα2 = interleukin 13 receptor alpha 2, IL = interleukin, CAR = chimeric antigen receptor, TME = tumor microenvironment, MDSC = myeloid-derived suppressor cells, NK = natural killer, GD2 = disialoganglioside, NK2D = natural killer group 2D.

## 6. Coexpression of Cytokine Receptors on CAR T-Cells

Another method to provide cytokine support for CAR cell-based therapy is to design CAR-encoding vectors to include cytokine receptors. These cytokine receptors can then either increase or decrease the response to a cytokine and, thus, enhance the functionality of modified CAR- or NK-cells. For example, Shum et al. genetically modified EphA2-CAR T-cells to express a constitutively active IL-7 cytokine receptor (C7R) (EphA2-CAR.C7R T-cells) [43] (Table 4). CR7 is a cytokine known to increase tumor-specific T-cells’ persistence and efficacy. The cytokine receptor was designed to be constitutively active and unresponsive to extracellular cytokines. Furthermore, C7R expression induced STAT5 phosphorylation but did not induce autonomous T-cell expansion, an important safety feature. EphA2-CAR.C7R T-cells induced complete regression of GBM U373 tumor xenografts at a cell dosage where unmodified EphA2-CAR T-cells were ineffective. Mechanistically, there was a correlation between improved antitumor activity and increased EphA2-CAR.C7R T-cell persistence.

One limitation of C7R is that it does not modulate neighboring immune cells. Swan et al. introduced IL-7 into EGFRvIII-targeted CAR T-cells to promote T-cell survival, proliferation, and increase memory of both CAR T-cells and neighboring immune cells [87] (Table 4). Fms-like tyrosine kinase receptor 3 ligand (Flt3L) is a cytokine and growth factor essential for dendritic cell (DC) function [88]. Flt3L has shown enhanced survival in preclinical studies [89,90] and promising preliminary outcomes in a clinical trial (NCT01811992) [91]. Therefore, Swan et al. generated EGFRvIII-CAR T-cells expressing IL-7 and/or Flt3L. Both IL-7 and IL7 with Flt3L enhanced intratumoral CAR T-cell abundance as well as conventional DCs and CD103+XCR1+ DCs in GBM xenografts, which are known to have migratory and antigen cross-presenting capabilities [87]. The generated CAR T-cells also showed greater antitumor activity, with a 67% improvement in overall survival in IL7-modified EGFRvIII-CAR T-cells and a 50% increase in survival within IL7/Flt3L EGFRvIII-CAR T-cells. Overall, IL-7 or IL-7 with Flt3L resulted in an augmented benefit compared to nonmodified EGFRvIII-CAR T-cells, most likely due to improved communication with neighboring immune cells.

One can also modify CAR T-cells with chemokine receptors to increase migration to the tumor and enhance therapeutic efficacy in GBM. For example, the chemokine IL-8 promotes the recruitment of tumor-associated neutrophils and MDSCs [92], angiogenesis [93], epithelial-mesenchymal transition [94], and cancer cell resistance, stemness, and metastatic potential [95]. Jin et al. incorporated the IL-8 receptors CXCR1 and CXCR2 into a CD70-targeted CAR T-cell [96] (Table 4). When delivered intravenously, CXCR1/2 modified CD70-CAR T-cells showed more remarkable CAR T-cell migration to the tumor site than their CD70-CAR T-cell-treated counterparts. They also showed superior antitumor activity, with nearly complete tumor clearance in GBM-bearing animals. The prevention of T-cell exhaustion and tumor rejection when rechallenged suggests modification with CXCR1/2 potentiates CAR T-cell efficacy and the formation of antitumor memory. Müller et al. used a similar construct to create CXCR4-modified EGFRvIII-CAR NK-cells [97] (Table 4). These enhanced CAR NK-cells demonstrated improved chemotaxis towards U87 GBM cells. In vivo, the CXCR4-modified EGFRvIII-CAR NK-cells showed superior cytotoxic activity and significantly improved survival in GBM-bearing mice compared to unmodified EGFRvIII-CAR NK-cells. Jin, Müller, and colleagues illustrate that incorporating chemokine receptors into CAR cells is feasible and can improve CAR treatment efficacy.

While some have focused on introducing stimulatory cytokine receptors into CAR constructs, others have focused on modifying constructs to reduce immunosuppressive signals to improve CAR functionality and efficacy. For example, transforming growth factor beta (TGF-β) contributes to tumor aggressiveness and dampens antitumor immune activity [98]. In GBM, Li et al. designed EGFRvIII-CAR T-cells that express a TGF-β receptor ectodomain (TGFRII ECD) that sequesters TGFβ [99](Table 4). These TGFβ-resistant EGFRvIII-CAR T-cells showed superior cytolytic activity against glioma cells when cocultured in vitro with TGF-β. When assessed in vivo, TGFβ-resistant EGFRvIII-CAR T-cells had a significantly greater reduction in tumor burden, prolonged the survival of xenografted mice, and significantly improved CAR T-cell infiltration into glioma sites compared to unmodified CAR T-cells. Additionally, TGFβ-resistant EGFRvIII-CAR T-cells also influenced the profile of infiltrated microglia to a more proinflammatory phenotype, with increased expression of CD86, CD11c, and MHCII and increased production of IFN-γ and TNF-α.

Similarly, Chaudhry and colleagues generated B7H3-targeted CAR-NK-cells with a TGF-β dominant-negative receptor (DNR) to target glioblastoma [100] (Table 4). The authors demonstrate that TGF-β can diminish B7H3-target NK-cells’ efficacy in targeting GBM tumors. Specifically, in a culture with U87 glioma cells, B7H3-CAR NK-cells only lysed 61% of tumor cells in the presence of TGF-β, compared to 90% of tumor cells when not in the presence of TGF-β. NK-cell activating receptors such as CD16 and NKG2D in the presence of TGF-β were significantly decreased, which may explain why the CAR NK-cells lose their efficacy. To negate the effect of TGF-β, the authors included a vector encoding the truncated human type II TGF-β receptor that functions to inhibit TGF-β signaling, which typically results in impaired NK-cell cytotoxicity. After five days of coculture with TGF-β, B7H3-CAR NK-cells with the TGF-β DNR maintained their ability to kill U87-gliomas and mitigate the downregulation of CD16 and NKG2D receptors. This effect was maintained with repetitive TGF-β stimulation. Since CAR T-cells are designed to engage and kill tumor cells and therapeutic efficacy has been partially diminished due to exhaustion, perhaps targeting immunosuppressive cytokines is a more worthwhile strategy.

A slightly different strategy than expressing receptors that increase or decrease the response of a cytokine is to use a chimeric cytokine receptor (CCR), or “switch receptor”. CCRs contain an ectodomain (extracellular component of the receptor) targeted to an inhibitory cytokine but are linked to an endodomain (intracellular component of the receptor) of a stimulatory cytokine. When a cytokine binds to these switch receptors, it elicits an entirely different response. While these CRC-modified CAR cells have not yet been tested within GBM, they have shown promising results in other cancers. For example, Mohammed et al. generated CAR T-cells with a CCR of extracellular IL-4R fused to the intracellular IL-7R for pancreatic cancer, converting the immunosuppressive response of IL-4 into an immunostimulatory pathway consistent with IL-7 activation [101]. Furthermore, Lange et al. created a CCR termed “GM18” consisting of a granulocyte-macrophage colony-stimulating factor (GM-CSF) ectodomain (a cytokine typically expressed post-CAR T-cell activation) and an IL18 receptor signaling endodomain [102]. Inclusion of this GM18 in both HER2-CAR T-cells for osteosarcoma and Eph2A-CAR T-cells for Ewing’s sarcoma led to enhanced killing of tumor cells and significantly greater ability to expand and produce cytokines compared to their unmodified counterparts. Overall, the success generated from CCRs in other solid tumors generates promise for their use in enhancing CAR T-cell therapy in glioblastoma and represents one uncharted avenue for future investigation.

**Table 4:** Including cytokine receptors into CAR constructs to enhance therapeutic efficacy.

Tumor Antigen	CAR Effector Cell	Study Type	Animal Model	Transgene	Delivery	TMZ, XRT, or Steroids?	Results	Reference
EphA2	T-cell	L	Mouse	IL-7R	IC	No	Enhanced antitumor activity of CAR T-cells, an increase in T-cell persistence, no exogenous ligand stimulation, or autonomous T-cell expansion decrease the risk of toxicity.	[43]
CD70	T-cell	L	Mouse	IL-8 R (CXCR1/CXCR2)	IC	XRT	Increase in CAR T-cell recruitment and persistence of therapy; obliterated tumors and prolonged survival, including at the end-stage of tumor progression and upon rechallenge.	[96]
EGFRvIII	T-cell	L	Mouse	TGFβ-trap	IV	No	Resistance of CAR T-cells to TGF-β suppression, greater reduction of tumor burden, prolonged survival, higher T-cell infiltration into the tumor site, and conversion of microglia to a more pro-inflammatory profile.	[99]
	NK-cell	L	Mouse	CXCR4	IV	No	Improvement in CAR NK-cell chemotaxis to U87-MG gliomas; improved survival in a U87 xenograft model	[97]
	T-cell	L	Mouse	IL-7/Flt3L	IC	XRT	Improved persistence of CAR T-cells, increased intratumoral abundance of CD103 + XCR1 + DCs	[87]
IL13Rα2	T-cell	L	Mouse	IL15Rα	IC	No	More significant anti-tumor activity compared to IL15-secreting CAR T-cell counterpart; modulation of TME to increase cytotoxic T-, NK-, and B-cells and reduce myeloid cells	[86]
	T-cell	L	Mouse	IL-15	IC	No	Transient improvement of CAR T-cells	[82]
B7H3	NK-cell	L	N/A	TGF-β	N/A	N/A	Improved CAR NK-cell resistance to TGF-β immunosuppression with improvement in NK-cell phenotype and cytolytic activity	[100]

Abbreviations: EphA2 = C = clinical trial, L = pre-clinical laboratory, IV = intravenous, IC = intracranial, IT = intraventricular, ephrin type-A receptor 2, IL = interleukin, CAR = chimeric antigen receptor, CD = cluster domain, CXCR = chemokine receptor, EGFRvIII = epidermal growth factor receptor variant III, TGFβ = transforming growth factor beta, NK = natural killer, Fms-related tyrosine kinase 3 ligand, DCs = dendritic cells, TME = tumor microenvironment.

## 7. Genetic Knock-Down of Cytokine Pathways in CAR T-Cells

Another potential strategy to modify cytokine signaling in CAR T-cells is to modulate cytokine-related genes. One method is to use clustered regularly interspaced short palindromic repeats (CRISPR)/Caspase 9 (Cas9) technology to knock down particular genes of interest. For example, Jung et al. targeted diacylglycerol kinase (DGK), an enzyme that phosphorylates diacylglycerol (DAG) to phosphatidic acid [103] (Table 5). Since DAG interacts with essential proteins within the CD3 pathway, they used CRISPR/Cas9 technology to knock out DGK, which increased the effector function and proliferation of CAR T-cells and made the cells more resistant to immunosuppressive cytokines such as TGFβ. Another application of gene technology to enhance CAR T-cell therapy has been the knockdown of transcription factor Ikaros and zinc finger transcription factor IKZF3, which usually suppress the expression of IL-2 and other cytokines in T-cells [104]. Zou et al. generated a CD133-specific CAR T-cell with IKZF3-knockout that demonstrated superior killing of glioma cells in vitro, as well as increased IFN-γ, IL-2, TNF-α, and GM-CSF cytokine levels compared to their unmodified counterparts [105] (Table 5). While using CRISPR/Cas9 technology to modify cytokine signaling pathways is a new addition to generating the most effective CAR cell-based therapy for glioblastoma, these results encourage the investigation into altering other cytokine pathways within other antiglioma CAR constructs.

An alternative to CRISPR/Cas9 genetic modification is to utilize short hairpin RNAs (shRNAs). While, to our knowledge, this technology has yet to be tested in glioblastoma, Kang et al. demonstrated the successful use of short hairpin RNA (shRNA) to knock down the IL-6 gene in CD19-CAR T-cells. These CAR T-cell populations significantly reduced CRS incidence in patients with acute lymphoblastic leukemia (ALL) [106]. Focusing on ICANS, Sterner et al. similarly sought to reduce toxicity associated with CAR T-cells [107]. The authors created GM-CSF knock-out CD19-CAR T-cells for leukemia using clustered regularly interspaced short palindromic repeats (CRISPR)/caspase 9 (Cas9) technology to eliminate GM-CSF secretion following CAR T-cell activation; these modified CAR T-cells diminished GM-CSF-induced neurotoxicity. Yi et al. developed a CAR T-cell therapy that knocked out GM-CSF and introduced transcripts encoding antibodies against IL-6 and IL-1 [108]. When tested in three patients with lymphoma or multiple myeloma, the modified CAR T-cell therapy showed no signs of neurotoxicity and a complete response in one patient with a low-moderate grade of CRS. Additionally, low levels of GM-CSF, IL-6, and IL-1γ were detected in all three patients. While this technology has yet to be applied in GBM, CRS, and ICANS occur at higher rates in blood-borne cancers, most likely due to the immunosuppressive effects of the TME within solid cancers [58]. With the impressive results seen for hematological malignancies, these approaches might also reduce side effects post-CAR T-cell therapy for solid tumors such as GBM.

**Table 5:** Enhancing cytokine production through genetic modification of CAR T-cells.

Tumor Antigen	CAR Effector Cell	Study Type	Animal Model	Transgene	Delivery	TMZ, XRT, or Steroids?	Results	Reference
EGFRvIII	T-cell	L	Mouse	DGK knock-out	IV	TMZ (c)	Improved effector functions of CAR T-cells and enhanced resistance to immunosuppressive cytokines (TGFβ).	[103]
CD133	T-cell	L	Mouse	IKZF3 knock-out	IV	No	Superior killing of glioma cells compared to their unmodified counterparts increased proinflammatory cytokines such as IL-2.	[105]

Abbreviations: EphA2 = C = clinical trial, L = preclinical laboratory, IV = intravenous, IC = intracranial, IT = intraventricular, DGK = diacylglycerol kinase; TGFβ = tumor growth factor beta, IKZF3 = IKAROS family zinc finger 3, IL = interleukin.

## 8. Conclusions

Providing cytokine support or manipulating cytokine signaling pathways has shown promise in preclinical GBM models to improve the effector function of CAR T- and NK-cells. Clearly, several of these approaches deserve evaluation in early-phase clinical studies. In this regard, prioritization will be critical since it is not feasible to test every approach. Our bias is that single-agent approaches should be evaluated due to the inherent complexities of administering multiple biotherapeutics. Should one select an approach that supports only genetically engineered immune cells (e.g., chimeric cytokine receptors) or an approach that directly influences bystander immune cells (e.g., secretory cytokines)? We believe that it will be impossible to definitively tease out differences in efficacy and safety profiles between both approaches in preclinical models, and thus recommend that both be evaluated in early-phase clinical studies for patients with GBM. In this regard, clinical trial design is key in mitigating the risk of adverse events. In addition to evaluating current approaches in early-phase clinical studies, further refinement of ‘cytokine enhanced’ immune cell approaches is needed, including manipulating multiple cytokine signaling pathways and efficiently regulating these once CAR immune cells have been infused. Finally, we are hopeful that carefully orchestrated correlative studies of early-phase clinical studies with ‘cytokine enhanced’ immune cells will guide further developments that will ultimately result in highly effective CAR T- and/or NK-cell products with a favorable safety profile for patients with GBM.
